# Trends in Antibiotic Susceptibility in Staphylococcus aureus in Boston, Massachusetts, from 2000 to 2014

**DOI:** 10.1128/JCM.01160-17

**Published:** 2017-12-26

**Authors:** Sanjat Kanjilal, Mohamad R. Abdul Sater, Maile Thayer, Georgia K. Lagoudas, Soohong Kim, Paul C. Blainey, Yonatan H. Grad

**Affiliations:** a Division of Infectious Diseases, Massachusetts General Hospital, Boston, Massachusetts, USA; b Harvard Medical School, Boston, Massachusetts, USA; c Department of Immunology & Infectious Diseases, Harvard T.H. Chan School of Public Health, Boston, Massachusetts, USA; d MIT Department of Biological Engineering, Broad Institute of Harvard and MIT, Cambridge, Massachusetts, USA; e Division of Infectious Diseases, Brigham and Women's Hospital, Boston, Massachusetts, USA; Medical College of Wisconsin

**Keywords:** *Staphylococcus aureus*, MRSA, antibiotic resistance, genomic epidemiology

## Abstract

The rate of infection by methicillin-resistant Staphylococcus aureus (MRSA) has declined over the past decade, but it is unclear whether this represents a decline in S. aureus infections overall. To evaluate the trends in the annual rates of infection by S. aureus subtypes and mean antibiotic resistance, we conducted a 15-year retrospective observational study at two tertiary care institutions in Boston, MA, of 31,753 adult inpatients with S. aureus isolated from clinical specimens. We inferred the gain and loss of methicillin resistance through genome sequencing of 180 isolates from 2016. The annual rates of infection by S. aureus declined from 2003 to 2014 by 4.2% (2.7% to 5.6%), attributable to an annual decline in MRSA of 10.9% (9.3% to 12.6%). Penicillin-susceptible S. aureus (PSSA) increased by 6.1% (4.2% to 8.1%) annually, and rates of methicillin-susceptible penicillin-resistant S. aureus (MSSA) did not change. Resistance in S. aureus decreased from 2000 to 2014 by 0.8 antibiotics (0.7 to 0.8). Within common MRSA clonal complexes, 3/14 MSSA and 2/21 PSSA isolates arose from the loss of resistance-conferring genes. Overall, in two tertiary care institutions in Boston, MA, a decline in S. aureus infections has been accompanied by a shift toward increased antibiotic susceptibility. The rise in PSSA makes penicillin an increasingly viable treatment option.

## INTRODUCTION

Antibiotic resistance is a major problem in the management of infection by Staphylococcus aureus, one of the most common bacterial pathogens (1). Methicillin-resistant S. aureus (MRSA) first appeared in the 1960s following the acquisition of the *mecA*-containing staphylococcal cassette chromosome *mec* (SCC*mec*). In the United States, the most widespread hospital-acquired MRSA clonal complex (CC) is CC5, which contains the multidrug-resistant strain known as USA100. MRSA infections acquired in the community are often due to the USA300 strain, which belongs to CC8, with evidence that this lineage is now also transmitted in the hospital setting (2, 3). The high prevalence of MRSA infections (4, 5) and the increased mortality, cost, and lengths of hospital stays of individuals infected with MRSA compared to those with methicillin-susceptible S. aureus (6, 7) focused research on describing MRSA epidemiology. In the United States and Europe, MRSA incidence peaked in 2005 and has been declining steadily since then (8–11).

In contrast with the extensive efforts to characterize MRSA, only a small number of studies have characterized the dynamics of all S. aureus subtypes, possibly due to the expectation that the prevalence of penicillin-susceptible S. aureus (PSSA) is extremely low (12). However, recent reports from diverse sites describe a rising or surprisingly high prevalence of PSSA (13–16). Whether this trend is associated with the decline in the prevalence of MRSA and how it impacts the overall rates of S. aureus infection are unclear. Furthermore, recent analyses of large data sets of S. aureus genome sequences have provided evidence that resistance is not permanent, but can be acquired and shed (17, 18). However, the generalizability of this observation is unclear.

To determine the overall trends in S. aureus antibiotic resistance and evaluate the hypothesis that the decline in the rates of MRSA infection has been accompanied by both an absolute and relative increase in PSSA incidence, we analyzed the electronic records of S. aureus infections in hospitalized patients from 2000 to 2014 at two tertiary care hospitals in Boston, MA. Through whole-genome sequencing of contemporary S. aureus invasive isolates, we tested the extent to which the trends are associated with specific S. aureus lineages and used phylogenomic methods to quantify the gains and losses of penicillin and methicillin resistance.

## MATERIALS AND METHODS

### Clinical data.

Clinical and microbiology data were obtained by medical record review of all inpatients admitted to the Brigham & Women's Hospital (BWH) and Massachusetts General Hospital (MGH) between 1 January 2000 and 31 December 2014 who were ≥18 years of age and had at least one nonsurveillance specimen from any site growing S. aureus. The clinical variables included age, sex, and Charlson comorbidity index (19) as determined by a review of ICD-9 codes. Clinical isolates were categorized as “blood” for those derived from blood cultures and therefore representing unambiguous infections and “nonblood” for those collected from other sites, which may include isolates not causally associated with infection. The isolates were labeled “community onset” if obtained within 48 h of admission and “hospital onset” thereafter. Isolates with susceptibility profiles identical to those of prior isolates from the same admission were considered duplicates and excluded.

### Clinical microbiology.

Clinical isolates were analyzed for susceptibility to penicillin (P), methicillin (M), erythromycin (E), clindamycin (C), levofloxacin (L), gentamicin, tetracycline, trimethoprim-sulfamethoxazole (TMP-SMX), rifampin, and vancomycin. The Clinical and Laboratory Standards Institute (CLSI) disk diameter method was utilized until 2008 at MGH and until 2009 at BWH, followed by the use of an automated broth microdilution method (Vitek2; bioMérieux) through 2014. Resistance to methicillin was inferred by testing for oxacillin and cefoxitin resistance. Two-step testing for inducible beta-lactamase activity in S. aureus isolates initially reported as susceptible to penicillin was performed using the nitrocefin disk followed by the zone-edge test (20) and was done at both hospitals after 2011; prior to that it was done upon clinician request. We included data for susceptibility to clindamycin only after 2010, since this was the first full year both hospitals performed inducible clindamycin resistance testing on all specimens automatically. We define an antibiogram type as the set of antibiotics to which an isolate is resistant. For example, “PME” signifies resistance to penicillin, methicillin, and erythromycin. A detailed description and timeline of the antimicrobial susceptibility testing protocols performed at each hospital is shown in Fig. S6 in the supplemental material. The susceptibility breakpoints were per CLSI guidelines over the study period; isolates that were “intermediate” by CLSI breakpoints were grouped with resistant isolates for all analyses. We categorized a specimen as PSSA if it was susceptible to penicillin and methicillin, MSSA if it was resistant to penicillin and susceptible to methicillin, and MRSA if it was resistant to both penicillin and methicillin.

### Statistical analyses.

We analyzed the rate of infections by S. aureus per 1,000 inpatient admissions and the mean number of antibiotics to which specimens are resistant. Annual changes in counts were adjusted for patient volume. All analyses were stratified by S. aureus subtype or antibiogram type and adjusted for age, sex, Charlson comorbidity index, type of isolate (blood versus nonblood), and onset of infection (community versus hospital). The analysis with antibiogram type excluded clindamycin in order to examine trends for the entire study period. Analyses were performed in R version 3.2.2 (21) on data pooled from both facilities. The tests of difference between subtypes or antibiogram types were 2-sided and comprised *t* tests for continuous variables, chi-squared tests with correction for multiple hypothesis testing for categorical variables, and the Wilcoxon-Mann-Whitney test for the Charlson comorbidity index. Linear and Poisson regressions (with patient volume as the offset when applicable) were used for multivariable adjustments of rate and count data, respectively, and 95% confidence intervals were calculated by the profile likelihood method in the R package MASS.

### Prospective specimen collection, sequencing, and analysis.

We collected a convenience sample of 180 nonduplicate isolates of S. aureus from patients ≥18 years of age who had specimens submitted to the BWH clinical microbiology laboratory between 1 January 2016 and 22 July 2016, representing 15% of all S. aureus isolates identified at BWH over this time period. The clinical characteristics of patients from this sample were similar to those of the retrospective patient cohort, with minor differences in the distributions of sex, the Charlson comorbidity index, and the onset of infection owing to small sample sizes (see Table S1). The isolates were processed into shotgun sequence libraries on a microfluidic platform using the Illumina Nextera protocol as described previously (22) and sequenced on an Illumina platform. We mapped reads to USA300 (GenBank no. NC_010079.1) using the Burrows-Wheeler alignment (BWA) tool (23), assembled genomes *de novo* with SPAdes (24), and annotated them with Prokka (25). We used Pilon (26) to identify single nucleotide polymorphisms (SNPs). Clonal complex, multilocus sequence types, and SCC*mec* types were assigned using eBURST (27) and online databases (28) (www.staphylococcus.net). We constructed a maximum likelihood phylogeny from the reference-based SNPs using RAxML (29) and ST152 (GenBank NZ_LN854556.1) as the outgroup. We inferred by parsimony the number of acquisitions and losses of SCC*mec* within sequence type 5 (ST5) and ST8 using Mesquite (http://mesquiteproject.org/), with N315 (GenBank NC_002745.2) as the reference genome for ST5 and USA300 (GenBank NC_010079.1) as the reference genome for ST8 isolates and with removal of Gubbins-predicted recombination blocks (30).

### Accession number(s).

All genome sequences have been deposited in the National Center for Biotechnology Information Sequence Read Archive (SRA) under SRA accession no. PRJNA380282. See the supplemental material for a detailed description of isolate preparation and bioinformatics analyses.

## RESULTS

### Clinical and microbiologic characteristics of S. aureus.

Our data set comprised records of 45,707 S. aureus isolates, including 22,799 MRSA, 18,154 methicillin-susceptible penicillin-resistant S. aureus (MSSA), and 4,754 PSSA isolates (Table 1). Patients with MRSA were older, had more comorbidities, and more often had the lungs as the sites of infection than patients with MSSA or PSSA. Patients with PSSA were older and had slightly more comorbidities than those with MSSA. Eighty percent of all specimens belonged to five antibiogram types (Table S1 in the supplemental material): two are MRSA (PMEL and PME), two are MSSA (PE and P), and one is PSSA (pansusceptible). Patients infected by PME S. aureus were significantly younger and had fewer comorbidities and a higher proportion of skin and soft tissue infection (SSI) isolates relative to all other antibiogram types. Patients with PMEL S. aureus were significantly older and had a higher proportion of lung isolates (Table S2). Less than 6% of isolates were resistant to gentamicin, tetracycline, TMP-SMX, and rifampin, and there were no isolates resistant to vancomycin.

**TABLE 1:** Demographic and microbiologic characteristics of patients and S. aureus subtypes

Subtype	No. of isolates	Mean patient age (yr [SD])	% female patients	CCI^*a*^ (mean [median])	Site of infection (%)				% community onset infections
					Blood	Lung	SSI	Other	
All S. aureus	45,707	58.8 (18.3)	41	2.8 (2)	16	34	28	22	62
MRSA	22,799	61.5 (18.1)	42	3.0 (2)	16	38	25	22	54
MSSA	18,154	55.6 (18.2)	40	2.4 (2)	17	30	31	22	69
PSSA	4,754	57.8 (18.1)	40	2.6 (2)	16	32	29	23	68
*P* value^*b*^									
MRSA vs MSSA		<0.0001	0.005	<0.0001	<0.0001				<0.0001
MRSA vs PSSA		<0.0001	0.12	<0.0001	<0.0001				<0.0001
MSSA vs PSSA		<0.0001	0.87	0.0005	0.0012				0.76

a CCI, Charlson comorbidity index; SSI, skin and soft tissue infection.

b Tests of difference are two-sided and comprised *t* tests for age, chi-squared tests for sex, site of infection, and onset, and a Mann-Whitney test for CCI.

### Trends in S. aureus infection.

**FIG 1:**
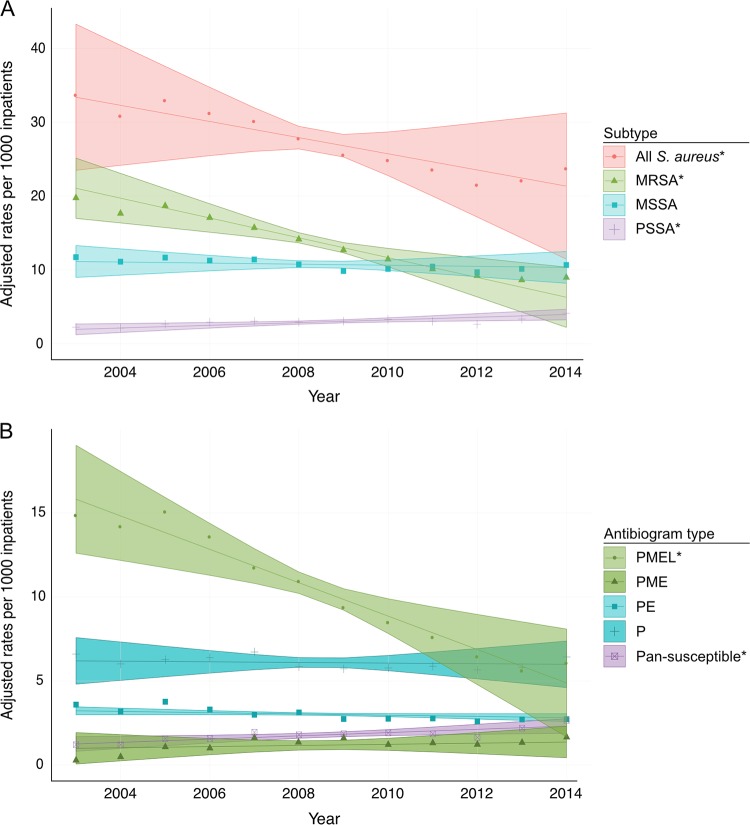
Rates of infections by S. aureus per 1,000 inpatients from 2003 to 2014 by subtype (A) and major antibiogram type (B). Estimates adjusted for age, sex, Charlson comorbidity index, type of clinical isolate (blood versus nonblood) and onset (community versus hospital). Lines represent model fits, shaded areas are 95% confidence intervals, and data points represent unadjusted rates. Asterisks indicate the trends are significant. The “other” category for antibiogram type was omitted for clarity.

After adjusting for age, sex, comorbidities, type of isolate, and onset of infection, the rates of infection from S. aureus were stable from 2000 to 2003, but subsequently declined annually from 2003 to 2014 by 4.2% (95% confidence interval [CI], 2.7% to 5.6%) (Fig. 1A and Table 2), from 33.4 to 21.3 infections per 1,000 inpatients. This pattern was driven predominantly by MRSA, which declined after 2003 by 10.9% per year (95% CI, 9.3% to 12.6%) from 21.1 to 6.3 infections per 1,000 inpatients over this time period. In contrast, over the 2003 to 2014 interval, PSSA increased by 6.1% annually (95% CI, 4.2% to 8.1%) from 1.9 to 4.0 infections per 1,000 inpatients. There was no change in the rates of MSSA over this time period. We evaluated the rates of resistance to erythromycin, clindamycin, and levofloxacin and observed significant declines for all three drugs in MRSA isolates and declines in levofloxacin and erythromycin resistance in PSSA and in MSSA isolates (see Fig. S1).

**TABLE 2:** Adjusted rates of inpatient infections of S. aureus by subtype and antibiogram type per 1,000 inpatients in 2003 and 2014

Subtype or antibiogram type	Rate of infections/1,000 inpatients (% [95% CI])^*b*^		Annual % change (95% CI) in counts^*b*^	*P* value
	2003	2014		
Subtype				
All S. aureus	33.4 (23.5–43.3)	21.3 (11.5–31.2)	−4.2 (−5.6 to −2.7)	<0.0001
MRSA	21.1 (17.0–25.1)	6.3 (2.2–10.4)	−10.9 (−12.6 to −9.3)	<0.0001
MSSA	11.1 (9.0–13.3)	10.3 (8.2–12.5)	−0.6 (−2.1 to 0.9)	0.43
PSSA	1.9 (1.2–2.7)	4.0 (3.2–4.7)	6.1 (4.2 to 8.1)	<0.0001
Antibiogram type^*a*^				
PMEL	15.8 (12.6–19.0)	4.9 (1.7–8.1)	−12.1 (−13.6 to −10.5)	<0.0001
PME	1.0 (0.1–1.9)	1.4 (0.4–2.3)	3.0 (−0.1 to 6.2)	0.06
PE	3.2 (3.0–3.5)	2.8 (2.6–3.1)	−1.4 (−3.2 to 0.5)	0.14
P	6.2 (4.8–7.6)	6.0 (4.6–7.4)	−0.3 (−2.3 to 1.8)	0.80
Pansusceptible	1.3 (0.8–1.7)	2.3 (1.9–2.8)	5.3 (3.1 to 7.5)	<0.0001
Other	3.9 (1.8–6.0)	6.0 (3.9–8.0)	3.3 (0.2 to 6.4)	0.04

a Antibiogram types exclude clindamycin. “Other” category includes all antibiograms not belonging to top 5 most common antibiogram types. PMEL, penicillin-, methicillin-, erythromycin-, and levofloxacin-resistant S. aureus; PME, penicillin-, methicillin-, and erythromycin-resistant S. aureus; PE, penicillin- and erythromycin-resistant S. aureus; P, penicillin-resistant S. aureus.

b Percentage estimates adjusted for age, sex, Charlson comorbidity index, type of clinical isolate (blood versus nonblood), and onset (community versus hospital).

The rates of the drug-resistant antibiogram type PMEL declined annually by 12.1% (95% CI, 10.5% to 13.6%), while those of the pansusceptible antibiogram type increased by 5.3% annually (95% CI, 3.1% to 7.5%) (Fig. 1B and Table 2). The rates did not change significantly for the two major MSSA types (PE and P). There was a nonsignificant trend toward an increase in the PME antibiogram type by 3.0% annually (95% CI, −0.1% to 6.2%). In the analysis including clindamycin for the interval 2010 to 2014, the trends were consistent between antibiogram types that included and excluded clindamycin (see Fig. S2).

### Changes in mean antibiotic resistance.

**FIG 2:**
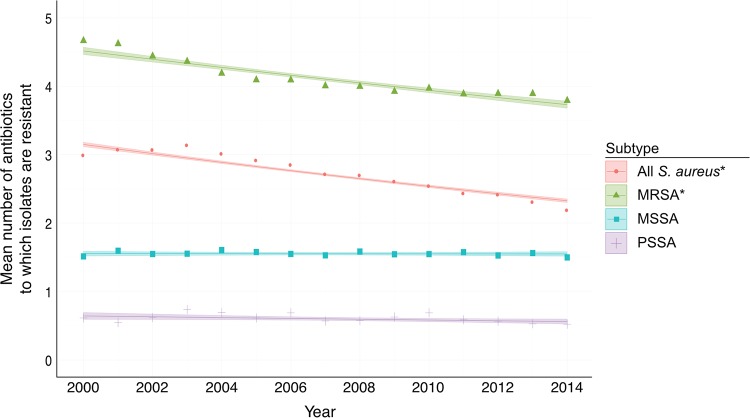
Mean resistances of S. aureus isolates from 2000 to 2014. Estimates adjusted for age, sex, Charlson comorbidity index, type of clinical isolate (blood versus nonblood), and onset (community versus hospital). Lines represent model fits, shaded areas are 95% confidence intervals, and data points represent unadjusted mean resistances. Asterisks indicate trends are significant.

Given the rise in PSSA and the decline in those for multidrug-resistant S. aureus, we tested whether the overall susceptibility of S. aureus changed during the study period. In 2000, an S. aureus infection was on average resistant to 3.2 antibiotics. By 2014, this decreased to 2.3 antibiotics (*P* < 0.0001 for the trend) (Fig. 2 and Table 3). When stratifying by subtype, we found that there was a decline in the mean resistance of MRSA isolates (4.5 antibiotics in 2000 to 3.7 antibiotics in 2014; *P* < 0.0001 for the trend), but there was no change in the average resistances of MSSA and PSSA isolates. When including clindamycin for the interval 2010 to 2014, there was an increase in overall resistance but no change in the time trend, except for a slightly greater decline in MRSA isolates (see Fig. S3). The declines occurred regardless of whether the isolates were strictly pathogenic (obtained from blood cultures) or if they were collected from other body sites (Fig. S3).

**TABLE 3:** Adjusted mean antibiotic resistances of S. aureus by subtype in 2000 and 2014

Subtype	Mean resistance (no. of antibiotics [95% CI])^*a*^		Absolute change (no. of antibiotics [95% CI])	*P* value
	2000	2014		
All S. aureus	3.2 (3.1–3.2)	2.3 (2.3–2.4)	−0.8 (−0.8 to −0.7)	<0.0001
MRSA	4.5 (4.5–4.6)	3.7 (3.7–3.8)	−0.9 (−1.0 to −0.8)	<0.0001
MSSA	1.6 (1.5–1.6)	1.5 (1.5–1.6)	0.0 (−0.1 to 0.1)	0.89
PSSA	0.6 (0.6–0.7)	0.6 (0.5–0.6)	−0.1 (−0.1 to 0.0)	0.05

a Estimates adjusted for age, sex, Charlson comorbidity index, type of clinical isolate (blood versus nonblood), and onset (community versus hospital).

### Population structure of contemporary S. aureus.

**FIG 3:**
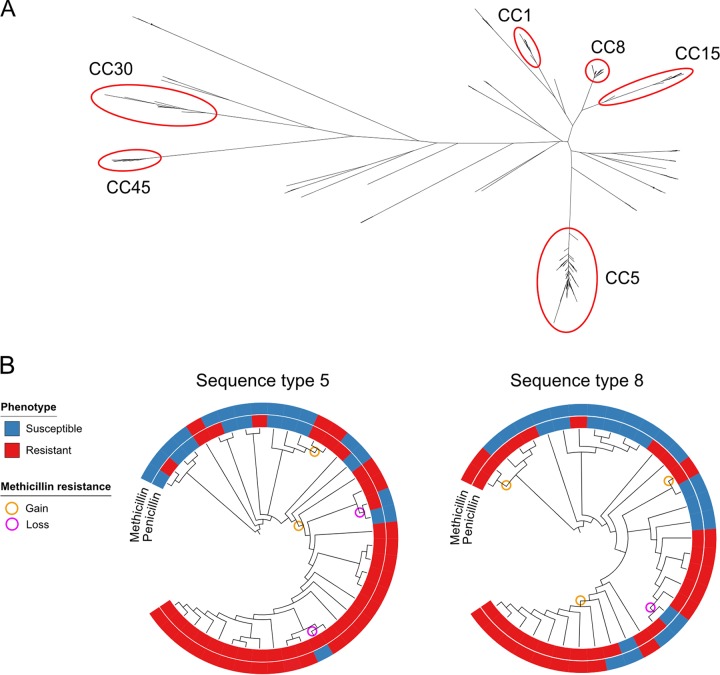
Phylogeny of contemporary S. aureus isolates and gain and loss of methicillin resistance at the strain level. (A) Unrooted maximum likelihood phylogeny of 180 S. aureus isolates obtained between 1 January 2016 and 22 July 2016. Select clonal complexes are identified in red circles. (B) Inference of ancestral presence or absence of methicillin resistance in sequence type 5 (CC5) and sequence type 8 (CC8) phylogenies (shown here as dendrograms) estimated using parsimony. Inner rings represent penicillin susceptibility, and outer rings represent methicillin susceptibility. Orange circles represent acquisition of methicillin resistance, and magenta circles represent loss of methicillin resistance. Branch lengths are intended to maximize visual clarity and are not proportional to genetic distance. CC, clonal complex.

Figure 3A illustrates the unrooted phylogeny for 180 clinical isolates (58 MRSA, 53 MSSA, and 69 PSSA isolates). The two most common genetic lineages in this sample were CC5 (*n* = 62) and CC8 (*n* = 43). Other clonal complexes with multiple specimens include CC1 (*n* = 14), CC15 (*n* = 11), CC30 (*n* = 7), and CC45 (*n* = 7). Isolates belonging to each of these CCs clustered together in the phylogeny, with the exception of sequence type 6 ([ST6] CC5) and ST72 (CC8) isolates, which were located on separate branches. Thirty-six isolates belonged to minor clonal complexes (each with ≤5 isolates per CC), and 1 isolate had three novel alleles at the multilocus sequence type (MLST) loci (see Fig. S4). MRSA isolates were limited to CC5 (39/62) and CC8 (19/38) (see Table S3). Of the PMEL isolates, 97% were CC5 and 94% of these were also resistant to clindamycin (PMECL). Of the PME isolates, 80% were CC8 and only 20% of these were resistant to clindamycin (Tables S1 and S3). MSSA and PSSA isolates and their corresponding antibiogram types were polyclonal. CC5 and CC8 isolates exhibited a wide range of antibiotic resistance phenotypes (Fig. 3B; see also Fig. S4). Notably, 23 of 62 isolates (37%) in the hospital-associated lineage CC5 and 24 of 43 (56%) in CC8 were MSSA or PSSA. On the basis of the inference from the phylogeny, we identified a gain and loss of *mecA* (Fig. 3B), indicating that penicillin and methicillin resistances are dynamic in S. aureus populations. In most cases, isolates with a loss of *mecA* had a complete loss of SCC*mec* and the adjacent arginine catabolic mobile element, if present (see Fig. S5). Overall, 2/19 (11%) of PSSA and MSSA isolates in sequence type 8 and 3/16 (19%) in sequence type 5 are inferred to represent isolates that derived from MRSA lineages.

## DISCUSSION

Over the past 15 years, the rates of MRSA in hospitalized patients at two tertiary care hospitals in Boston, MA, have declined markedly, the rates of PSSA have increased, and the overall rates of S. aureus infections have declined slightly (Fig. 1). Combined with the decreased resistance to other antibiotics, S. aureus infections on average have become more antibiotic susceptible over the past 10 years. Further, the observed decline in the rates of MRSA infections does not reflect a decline in all MRSA strains; our results suggest that the decline is primarily in the PMEL antibiogram type, primarily represented by CC5.

The observation that penicillin and methicillin resistance are gained and lost at the strain level represents a departure from the dogma that population-level resistance reflects lineages with stable resistance phenotypes. Similar findings were noted in a recent study from France that showed a loss of methicillin resistance in CC30 (18).

One potential explanation for the trend of increasing S. aureus antibiotic susceptibility is a shift in antibiotic pressures. The declines in the use of narrow-spectrum beta-lactams such as oxacillin and penicillin G since 2000 and in the inpatient use of first-generation cephalosporins since 2006 (31) may select against hospital acquired (HA) MRSA in favor of PSSA, but do not on their own explain the stable incidence of community acquired (CA) MRSA. We also note the decline in levofloxacin resistance in all types of S. aureus is occurring over a time period when the use of levofloxacin declined significantly in inpatient settings (31).

There are several limitations to our study. First, the testing for inducible beta-lactamase production was not routinely performed prior to 2011, raising the possibility that specimens reported as penicillin susceptible from this time period were in fact penicillin resistant. However, an underestimate of penicillin resistance prior to 2011 would indicate that the increasing rates of PSSA infections that we report are an underestimate of the true increase. Second, in the absence of genotyping of historical specimens, the evidence that the decline in MRSA has occurred disproportionately within CC5 relies on the consistency in the demographic and microbiologic characteristics of antibiogram types between our retrospective and prospective cohorts. However, there was little difference in the clinical characteristics of our prospective sample and our retrospective sample. Furthermore, the overrepresentations of PMECL isolates in CC5 and PME isolates in CC8 suggest that these antibiogram types are rough proxies for lineage. Lastly, the generalizability of our results may be limited, as this analysis is restricted to inpatients admitted to 2 hospitals in the same geographic area. However, the rates of decline of MRSA in our study are similar to the rate of decline from a nationally representative study (8), and the decline in the erythromycin, clindamycin, and levofloxacin antibiogram type was recently noted in a sample of MRSA isolates taken from 20 hospitals across the United States (2). Furthermore, recent reports from geographically diverse institutions note increased rates of PSSA infection (13, 14, 16). A major strength of this study was the unbiased analysis of the overall S. aureus population, whereas most prior studies have examined only the MRSA subpopulation. This has yielded a more complete picture of the clonal dynamics of this highly adaptable pathogen.

The decline in antibiotic resistance in S. aureus over the past 10 years runs counter to the prevailing paradigm of an inexorable rise of multidrug resistance among human pathogens. Defining the forces driving the decline will be a critical task to guide efforts to control S. aureus infection and antibiotic resistance. The increasing incidence of PSSA should prompt an evaluation of broader geographic trends along with a reevaluation of the use of penicillin for the treatment of infections due to S. aureus.

## Supplementary Material

Supplemental material
