# Supplementary material for: Trends in Antibiotic Susceptibility in Staphylococcus aureus in Boston, Massachusetts, from 2000 to 2014
Source: J Clin Microbiol. 2017 Dec 26;56(1):e01160-17. doi: 10.1128/JCM.01160-17 (PMC5744217; doi:10.1128/JCM.01160-17)
Supplement: Supplemental material [file supp_56_1_e01160-17__index.html]

Trends in Antibiotic Susceptibility in Staphylococcus aureus in Boston, Massachusetts, from 2000 to 2014 — Supplemental material 

# Trends in Antibiotic Susceptibility in Staphylococcus aureus in Boston, Massachusetts, from 2000 to 2014

## Supplemental material

- Supplemental file 1 -

  Tables S1 (Characteristics of retrospective and prospective samples by subtype and antibiogram type), S2 (Demographic and microbiologic characteristics of *S. aureus* antibiogram types), and S3 (Distribution of clonal complex by subtype and antibiogram type); Fig. S1 (Adjusted rates of erythromycin, clindamycin, and levofloxacin resistance in *S. aureus*), S2 (Adjusted rates of *S. aureus* by major antibiogram type, excluding or including clindamycin, 2010 to 2014), S3 (Mean resistance in *S. aureus*, excluding or including clindamycin and comparing blood and nonblood isolates), S4 (Complete phylogeny of *S. aureus* isolates), S5 (Genetic basis for loss of methicillin resistance in ST5/CC5 and ST8/CC8), and S6 (Antibiotic susceptibility testing protocols for *S. aureus*, 2000 to 2014); and Supplemental methods (Detailed sample preparation for microfluidics platform and bioinformatic analyses)

  PDF, 1.4M
