# Supplementary material for: Trends in Antibiotic Susceptibility in Staphylococcus aureus in Boston, Massachusetts, from 2000 to 2014
Source: J Clin Microbiol. 2017 Dec 26;56(1):e01160-17. doi: 10.1128/JCM.01160-17 (PMC5744217; doi:10.1128/JCM.01160-17)
Supplement: Supplemental material [file JCM.01160-17_zjm999095782s1.pdf]

Supplemental material

Table / Figure	Title	Pg
Table S1	Characteristics of retrospective and prospective samples by subtype and antibiogram types.	2
Table S2	Demographic and microbiologic characteristics of *S. aureus* antibiogram types.	4
Table S3	Distribution of clonal complex by subtype and antibiogram types.	5
Figure S1	Adjusted rates of erythromycin, clindamycin and levofloxacin resistance in *S. aureus*.	6
Figure S2	Adjusted rates of *S. aureus* by major antibiogram type, excluding and including clindamycin, 2010 to 2014.	7
Figure S3	Mean resistance in *S. aureus* excluding and including clindamycin and comparing blood and non-blood isolates.	8
Figure S4	Complete phylogeny of *S. aureus* isolates.	9
Figure S5	Genetic basis for loss of methicillin resistance in ST5 / CC5 and ST8 / CC8.	10
Figure S6	Antibiotic susceptibility testing protocols for *Staphylococcus aureus* 2000 – 2014.	11
Supplementary Methods	Detailed sample preparation for microfluidics platform and bioinformatic analyses.	12

	Retrospective	Prospective	p-value[c]
**Sample size – n (%)**			
All *S. aureus*	45 707 (100)	180 (100)	
Subtype			
MRSA	22 799 (50)	58 (32)	
MSSA	18 154 (40)	53 (29)	
PSSA	4754 (10)	69 (38)	
Antibiogram type[a]			
PMEL	16 720 (37)	36 (20)	
PME	1715 (4)	15 (8)	
PE	5041 (11)	17 (9)	
P	10 359 (23)	26 (14)	
Pan-susceptible	2889 (6)	56 (31)	
Other[b]	8983 (20)	30 (17)	
**Mean age - Years (SD)**			
All *S. aureus*	58.8 (18.3)	55.5 (17.1)	**0.02**
Subtype			
MRSA	61.5 (18.1)	58.3 (16.0)	0.17
MSSA	55.6 (18.2)	52.0 (18.3)	0.15
PSSA	57.8 (18.1)	55.9 (16.8)	0.39
Antibiogram type[a]			
PMEL	63.8 (17.3)	62.0 (13.8)	0.54
PME	46.2 (16.7)	48.7 (18.2)	0.56
PE	54.3 (18.0)	49.4 (18.9)	0.26
P	55.5 (17.9)	52.8 (19.0)	0.44
Pan-susceptible	57.0 (17.8)	55.1 (16.8)	0.44
Other[b]	58.8 (18.6)	57.8 (16.2)	0.76
**Gender - % female**			
All *S. aureus*	41	49	0.04
Subtype			
MRSA	41	45	0.71
MSSA	40	66	**0.0002**
PSSA	40	39	0.94
Antibiogram type[a]			
PMEL	42	36	0.58
PME	43	73	**0.03**
PE	42	76	**0.01**
P	39	62	**0.03**
Pan-susceptible	38	41	0.69
Other[b]	42	40	1.00
**CCI – Mean (Median)**			
All *S. aureus*	2.8 (2)	2.0 (1)	**8×10^{-6}**
Subtype			
MRSA	3.0 (2)	2.1 (1)	**0.007**
MSSA	2.5 (2)	1.6 (0)	**0.0006**
PSSA	2.6 (2)	2.3 (1)	0.21
Antibiogram type[a]			
PMEL	3.2 (2)	2.6 (2)	0.25
PME	1.6 (1)	0.5 (0)	**0.04**
PE	2.5 (2)	2.1 (0)	0.05
P	2.3 (1)	0.9 (0)	**0.001**
Pan-susceptible	2.4 (2)	2.3 (1)	0.40
Other[b]	2.9 (2)	2.6 (2)	0.44

(continued on next page)

	Retrospective	Prospective	p-value[c]
**Type of isolate - % Blood**			
All *S. aureus*	16	16	0.92
Subtype			
MRSA	16	14	0.86
MSSA	17	13	0.58
PSSA	16	19	0.51
Antibiogram type[a]			
PMEL	14	11	0.81
PME	14	13	1.00
PE	23	12	0.39
P	14	15	0.77
Pan-susceptible	14	18	0.43
Other[b]	20	20	1.00
**Onset - % Community**			
All *S. aureus*	62	75	**0.0003**
Subtype			
MRSA	54	59	0.60
MSSA	69	83	**0.03**
PSSA	68	82	0.02
Antibiogram type[a]			
PMEL	54	47	0.50
PME	74	87	0.38
PE	68	82	0.30
P	69	88	**0.03**
Pan-susceptible	69	80	0.13
Other[b]	59	77	0.06
**Clindamycin resistance[d] – n (%)**			
All *S. aureus*	10378 (46)	60 (33)	**0.001**
Subtype			
MRSA	7583 (73)	40 (69)	0.60
MSSA	2096 (22)	12 (23)	1.00
PSSA	699 (24)	8 (12)	**0.02**
Antibiogram type[a]			
PMEL	3433 (85)	34 (94)	0.16
PME	121 (15)	3 (20)	0.88
PE	996 (62)	10 (59)	0.98
P	23 (1)	0 (0)	0.41
Pan-susceptible	3 (0)	1 (2)	1.00
Other[b]	1303 (50)	12 (40)	0.38

Table S1: Characteristics of retrospective and prospective samples by subtype and antibiogram types.

Abbreviations: MRSA, methicillin resistant *S. aureus*; MSSA, methicillin susceptible / penicillin resistant *S. aureus*; PSSA, methicillin and penicillin susceptible *S. aureus*; PMEL penicillin, methicillin, erythromycin and levofloxacin resistant *S. aureus*; PME, penicillin, methicillin and erythromycin resistant *S. aureus*; PE, penicillin and erythromycin resistant *S. aureus*; P, penicillin resistant *S. aureus*; p, p-value; CCI, Charlson comorbidity index; IQR, inter-quartile range

[a]Antibiogram types exclude clindamycin.

[b]'Other' category includes all antibiograms not belonging to top 5 most common antibiogram types.

[c]Tests of difference are t test for age; Mann-Whitney test for CCI; chi-squared test for gender and clindamycin resistance; Fisher's exact test for type of isolate and onset.
[d]For 2010 through 2014 only

	n	Mean age Years (SD)	Gender % Female	CCI Mean (Median)	Site of infection % Blood	% Lung	% SSI	% Other	Onset % Community
All *S. aureus*	45 707	58.8 (18.3)	41	2.8 (2)	16	34	28	22	62
PMEL	16 845	63.8 (17.3)	42	3.2 (2)	14	39	23	24	54
PME	1719	46.2 (16.7)	43	1.6 (1)	14	14	54	18	74
PE	5057	54.3 (18.0)	42	2.5 (2)	23	27	30	20	68
P	10 389	55.5 (17.9)	39	2.3 (1)	14	31	32	23	69
Pan-Susceptible	2907	57.0 (17.8)	38	2.4 (2)	14	32	31	23	69
Other	9407	58.8 (18.6)	42	2.9 (2)	20	34	25	22	59
p-value[a]									
PMEL v PME		<0.0001	0.57	<0.0001		<0.0001			<0.0001
PMEL v PE		<0.0001	1	<0.0001		<0.0001			<0.0001
PMEL v P		<0.0001	<0.0001	<0.0001		<0.0001			<0.0001
PMEL v Pan-S		<0.0001	<0.0001	<0.0001		<0.0001			<0.0001
PMEL v Other		<0.0001	0.56	<0.0001		<0.0001			<0.0001
PME v PE		<0.0001	0.61	<0.0001		<0.0001			<0.0001
PME v P		<0.0001	<0.0001	<0.0001		<0.0001			<0.0001
PME v Pan-S		<0.0001	0.0007	<0.0001		<0.0001			0.003
PME v Other		<0.0001	0.4	<0.0001		<0.0001			<0.0001

Table S2: Demographic and microbiologic characteristics of *S. aureus* antibiogram types.

Abbreviations: PMEL penicillin, methicillin, erythromycin and levofloxacin resistant *S. aureus*; PME, penicillin, methicillin and erythromycin resistant *S. aureus*; PE, penicillin and erythromycin resistant *S. aureus*; P, penicillin resistant *S. aureus*; Pan-S, pan-susceptible *S. aureus*, p, p-value; CCI, Charlson comorbidity index.

[a]Tests of difference are two-sided and comprised of t-test for age; chi-squared test for gender, site of infection and onset; Mann-Whitney test for CCI.

	Clonal complex						
	1	5	8	15	30	45	Other
**Sample size**							
Subtype							
MRSA	0	39	19	0	0	0	0
MSSA	3	9	11	9	5	2	14
PSSA	11	14	13	2	2	5	22
Antibiogram type							
PMEL	0	35	1	0	0	0	0
PME	0	3	12	0	0	0	0
PE	1	1	4	2	1	1	7
P	1	4	5	5	4	1	6
Pan-susceptible	10	10	8	2	1	5	20
Other	2	9	13	2	1	0	3
**Column %**							
Subtype							
MRSA	0	63	44	0	0	0	0
MSSA	21	15	26	82	71	29	39
PSSA	79	23	30	18	29	71	61
Antibiogram type							
PMEL	0	56	2	0	0	0	0
PME	0	5	28	0	0	0	0
PE	7	2	9	18	14	14	19
P	7	6	12	45	57	14	17
Pan-susceptible	71	16	19	18	14	71	56
Other	14	15	30	18	14	0	8
**Row %**							
Subtype							
MRSA	0	67	33	0	0	0	0
MSSA	6	17	21	17	9	4	26
PSSA	16	20	19	3	3	7	32
Antibiogram type							
PMEL	0	97	3	0	0	0	0
PME	0	20	80	0	0	0	0
PE	6	6	24	12	6	6	41
P	4	15	19	19	15	4	23
Pan-susceptible	18	18	14	4	2	9	36
Other	7	30	43	7	3	0	10

Table S3: Distribution of clonal complex by subtype and antibiogram types.

Abbreviations: MRSA, methicillin resistant *S. aureus*; MSSA, methicillin susceptible / penicillin resistant *S. aureus*; PSSA, methicillin and penicillin susceptible *S. aureus*; PMEL penicillin, methicillin, erythromycin and levofloxacin resistant *S. aureus*; PME, penicillin, methicillin and erythromycin resistant *S. aureus*; PE, penicillin and erythromycin resistant *S. aureus*; P, penicillin resistant *S. aureus*

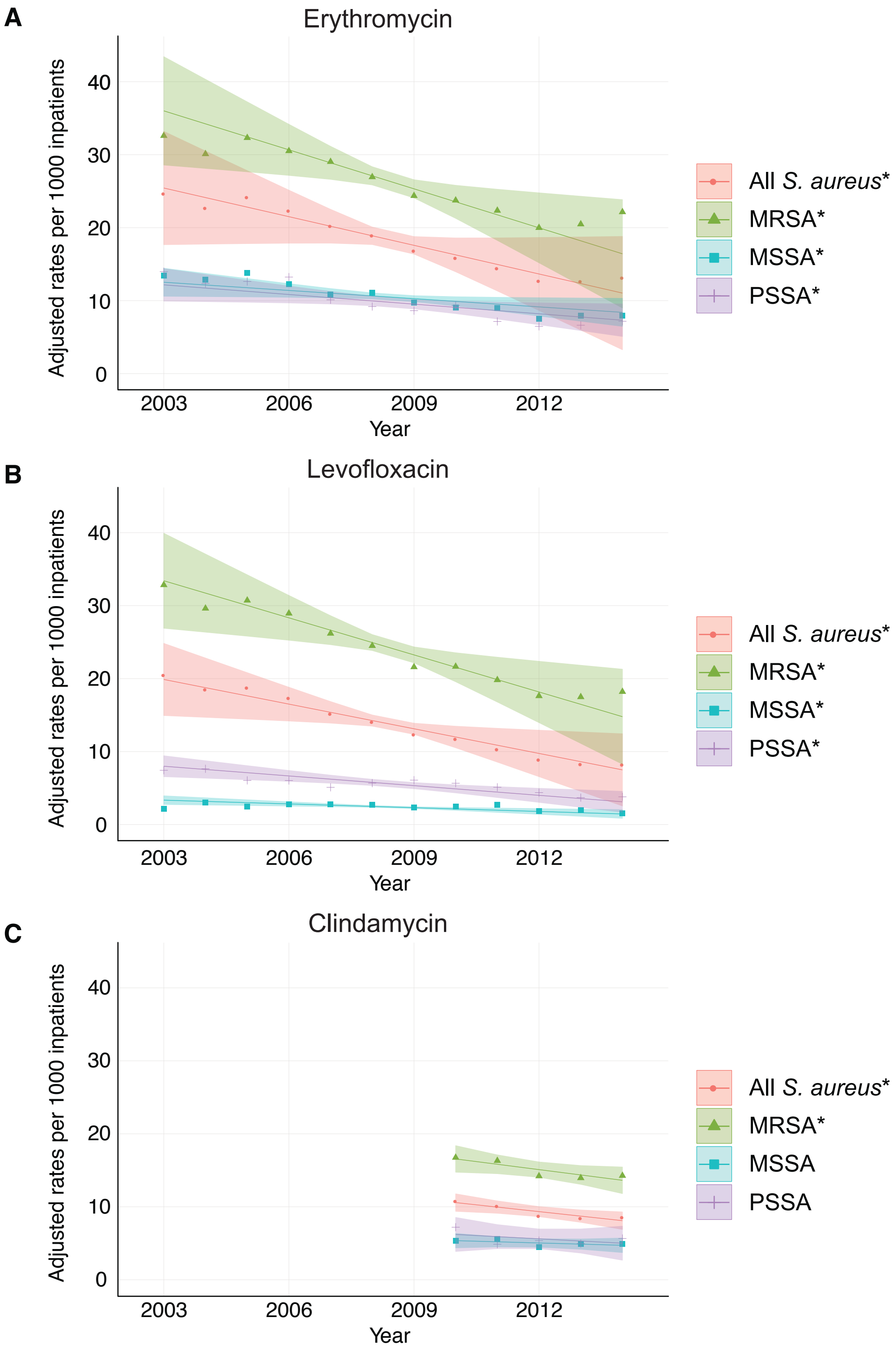

Figure S1: Adjusted rates of erythromycin, clindamycin and levofloxacin resistance in *S. aureus*. Rates of resistance of *S. aureus* per 1000 inpatients from 2003 to 2014 for (A) erythromycin and (B) levofloxacin and (C) from 2010 to 2014 for clindamycin. Estimates adjusted for age, gender, Charlson Comorbidity Index, type of clinical isolate (blood versus non-blood) and onset (community versus hospital). Estimates for (C) adjusted for year only due to small sample size. Line represents model fit, shaded areas are 95% confidence intervals and data points represent unadjusted rates. Asterisk indicates trend is significant.

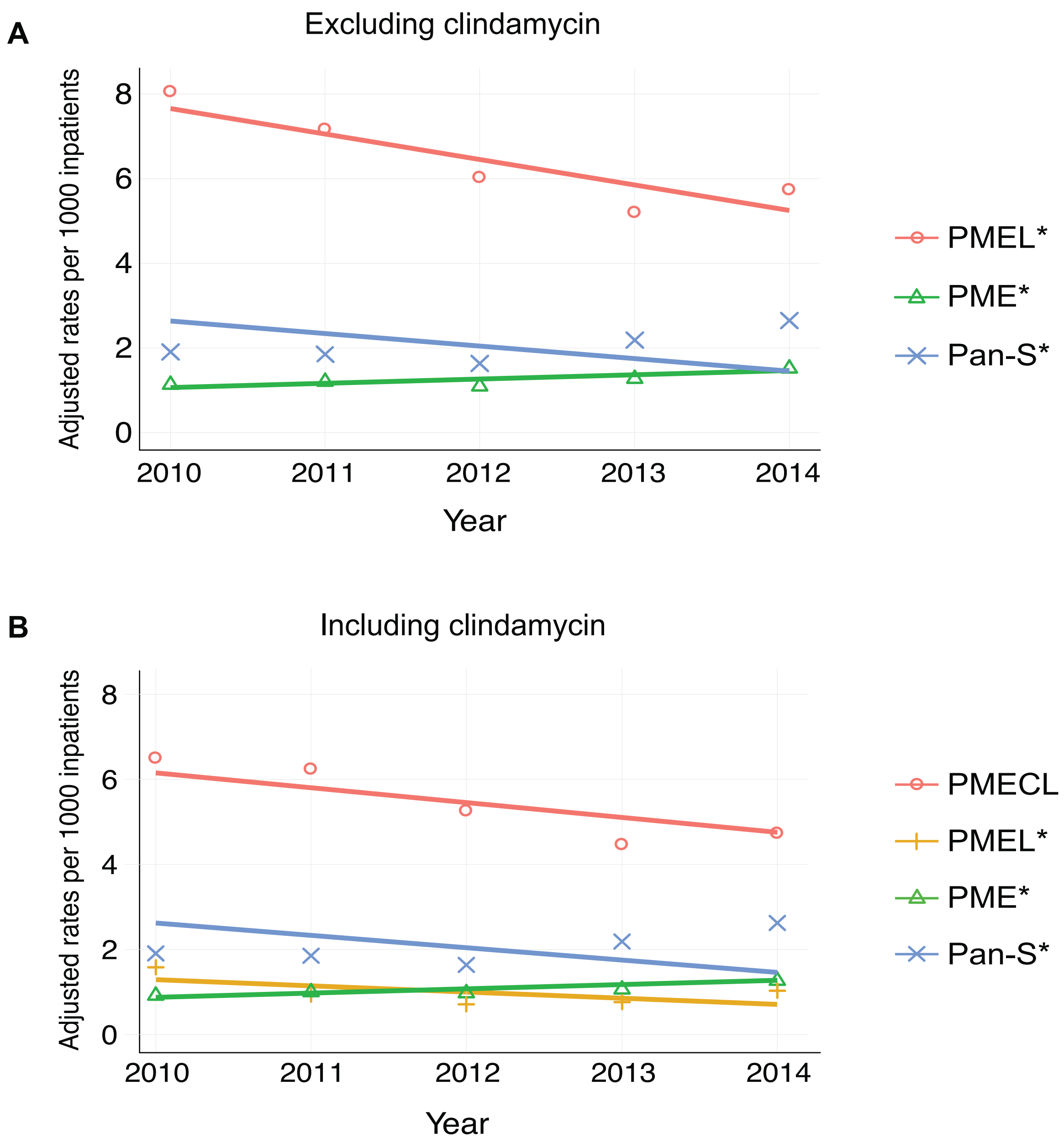

Figure S2: Adjusted rates of *S. aureus* by major antibiogram type (A) excluding and (B) including clindamycin, 2010 to 2014. Rates of inpatient infection of *S. aureus* by antibiogram type per 1000 inpatients from 2010 to 2014, excluding and including clindamycin. Estimates adjusted for year, age, and Charlson Comorbidity Index. Line represents model fit and data points represent unadjusted rates. Asterisk indicates trend is significant.

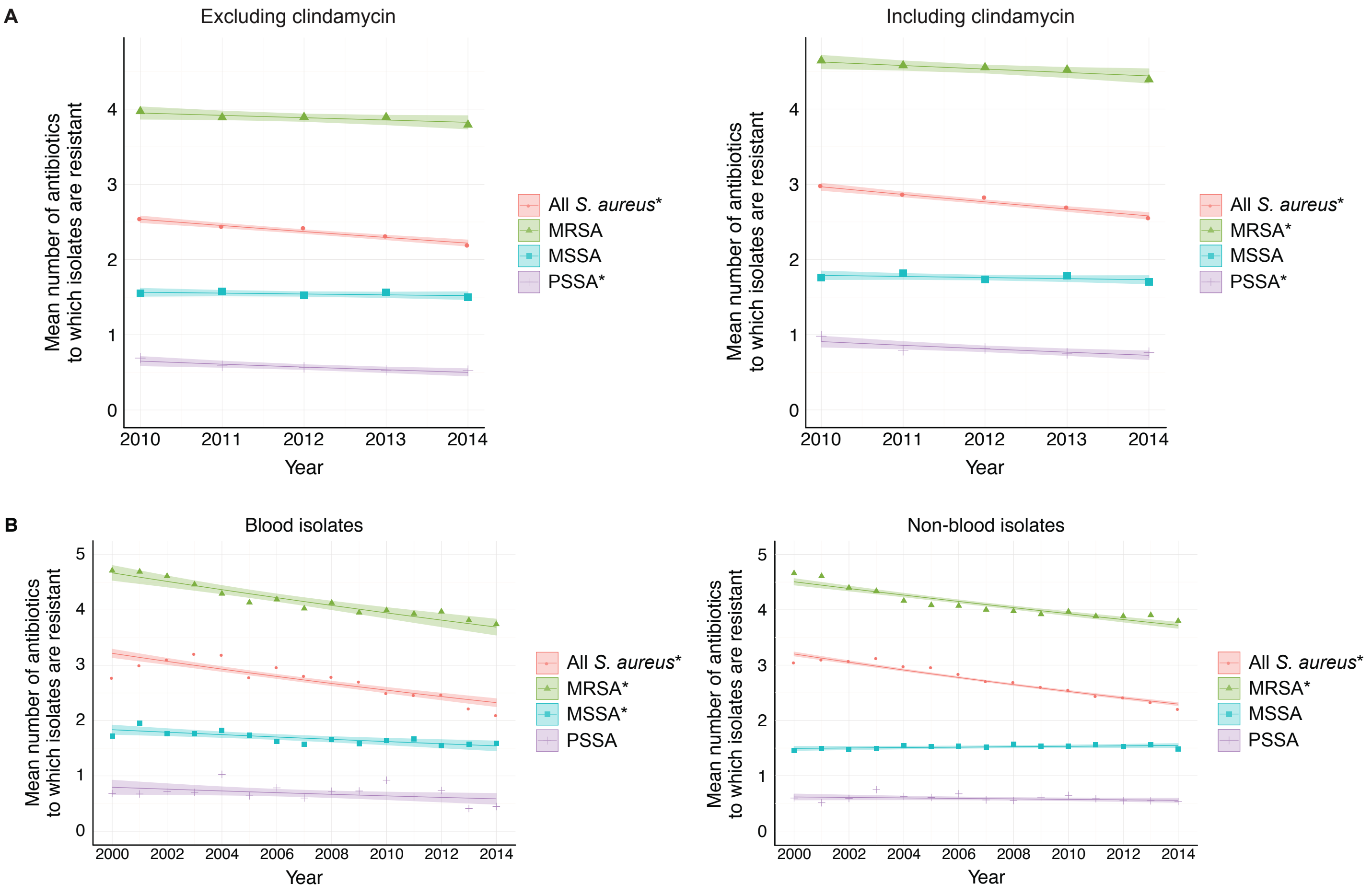

Figure S3: Mean resistance in *S. aureus* (A) excluding and including clindamycin and (B) comparing blood and non-blood isolates. Estimates for (A) adjusted for age, gender, Charlson Comorbidity Index, type of clinical isolate (blood versus non-blood) and onset (community versus hospital). Estimates for (B) do not include clindamycin and are adjusted for age, gender, Charlson Comorbidity Index, and onset (community versus hospital). Line represents model fit, shaded areas are 95% confidence intervals and data points represent unadjusted rates. Asterisk indicates trend is significant.

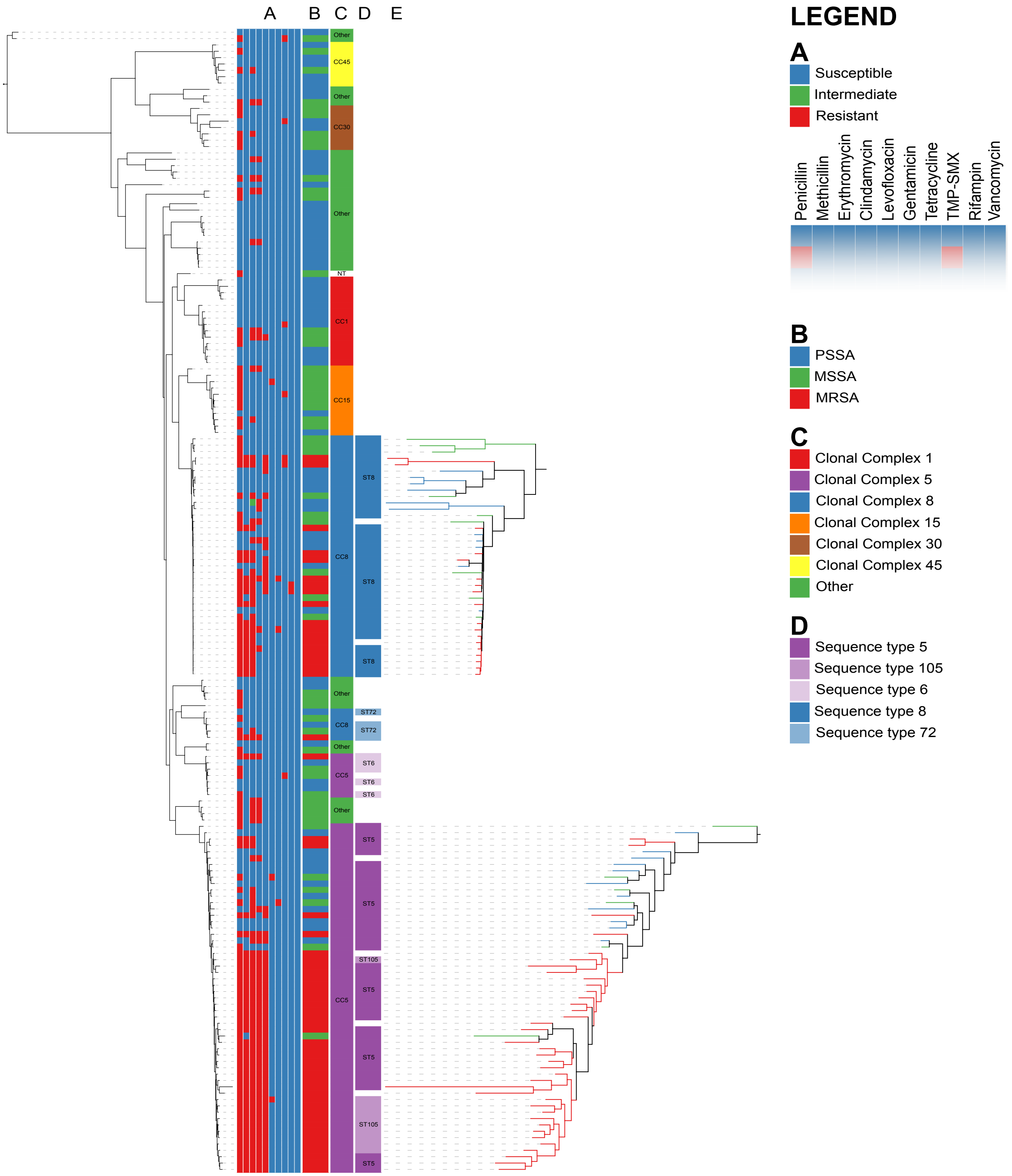

Figure S4: Complete phylogeny of contemporary *S. aureus* isolates. (A) Full resistance phenotype of sequenced *S. aureus* isolates; (B) *S. aureus* subtype; (C) Clonal complex; (D) Sequence types for CC5 and CC8; (E) Close up of ST5 (CC5), ST105 (CC5) and ST8 (CC8) isolates with branches colored by *S. aureus* subtype.

Abbreviations: MRSA, methicillin resistant *S. aureus*; MSSA, methicillin susceptible and penicillin resistant *S. aureus*; PSSA, methicillin and penicillin susceptible *S. aureus*; CC, clonal complex; ST, sequence type, NT, non-typeable.

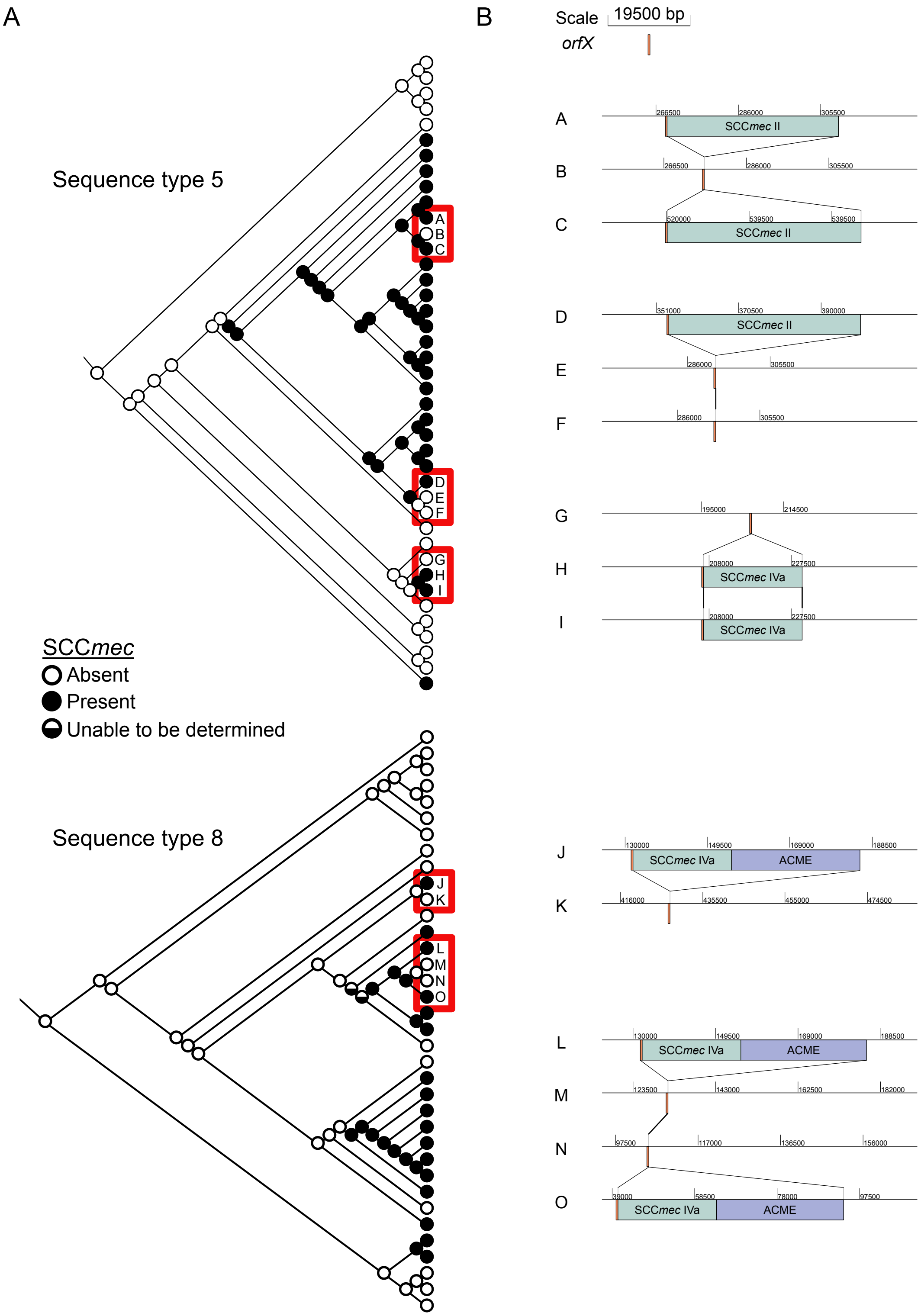

Figure S5: Genetic basis for loss of methicillin resistance in ST5 / CC5 and ST8 / CC8 (A) Maximum likelihood tree with ancestral presence or absence of SCC*mec* in ST5 and ST8, estimated using parsimony; (B) Close up of mechanism of SCC*mec* loss or gain in select isolates highlighted in panel A.

Abbreviations: ST, sequence type; CC, clonal complex; SCC, Staphylococcal cassette chromosome; ACME, arginine catabolic mobile element.

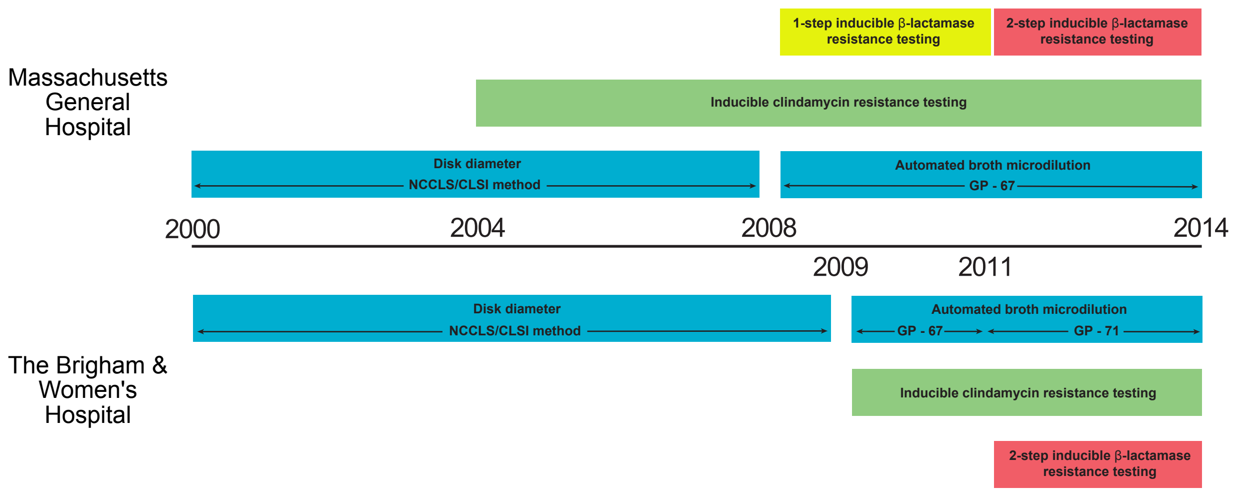

Figure S6: Antibiotic susceptibility testing protocols for *Staphylococcus aureus* 2000 – 2014. Vitek commercial antibiotic susceptibility testing cards shown under automated broth microdilution. Two-step testing in all *S. aureus* isolates for inducible beta-lactamase activity against penicillin was performed at both hospitals after 2011 and prior to that was done upon clinician request. Inducible resistance to clindamycin was performed at both hospitals on all isolates routinely after 2010. Resistance to methicillin was inferred by testing isolates against oxacillin and additionally against cefoxitin.

Supplementary Methods

Detailed sample preparation for microfluidics platform and bioinformatic analyses.

DNA extraction and library construction

DNA libraries were prepared from bacterial cultures using a previously described automated microfluidic sample preparation device (1). This device was used to minimize the reagent cost and hands-on time for the 180 total samples prepared.

S. aureus isolates were obtained on frozen beads from the BWH clinical microbiology laboratory and grown in Brain-Heart infusion media for 18-24 hours at 37 degrees Celsius and shaken at 220 rpm. Cultures were spun down at 5,000 rpm for 5 minutes and the supernatant was discarded. Pellets were resuspended in 1 ml of phosphate buffered saline. The lysis enzyme mix for genomic DNA isolation was modified from Kim et al by adding 2 μL lysostaphin (2.5 mg/mL) instead of 2 μL of buffer to accommodate *S. aureus*.

The microfluidics device takes 2 μL aliquots of cells as input and subsequently performs enzymatic cellular lysis, genomic DNA purification, tagmentation to construct DNA sequencing libraries, and library cleanup, size selection, and elution steps. All mixing and elution steps are performed in the device, using valves in the two-layer microfluidic architecture. DNA capture and cleanup is achieved using solid phase reversible immobilization (SPRI) beads inside the device. The tagmentation reaction is performed following the Illumina Nextera protocol using the Tagment DNA Enzyme (Illumina), as described in Kim et al (1).

Amplification and barcoding PCR

The DNA library was eluted from the device (8 μL) and combined with 10 μL of NEBNext High Fidelity 2X PCR MasterMix, 1 μL forward primer, and 1 μL reverse primer (obtained from the Broad Institute Genomics Platform). We performed PCR barcoding and amplification using the following protocol: 72C for 3 minutes, 98C for 30 seconds, 17 cycles of [98C for 10 seconds, 60C for 30 seconds, 72C for 30 seconds], and 72C for 5 minutes. The barcoded DNA was purified suing SPRI beads.

Sample pooling and sequencing

Barcoded libraries were quantified with Quant-iT (ThermoFisher) and pooled at equal concentrations. DNA sequencing was performed on the IIllumina MiSeq (2x150 and 2x75 cycle runs). Samples with less than 20X coverage and contigs less than 500bp in length were discarded.

Genomic analysis

Genome assemblies were generated with SPAdes v3.9 (2). Isolate sequence type (ST) and the corresponding clonal complex (CC) was determined using the PubMLST database (https://pubmlst.org/saureus/) (3) and eBURST (http://saureus.mlst.net/eburst/) (4). Assembled contigs from ST5 and ST8 isolates were re-ordered using ABACAS(5) with respect to reference genomes N315 (NC_002745.2) and TCH1516 (NC_010079.1), depending on isolate sequence types. Genome annotations were generated using Prokka v1.11 (6). Whole genome alignments were produced using ProgressiveMauve (7) and pairwise BlastN (8). The SCC*mec* cassette was visualized with the Artemis Comparison Tool (9) and typed using in-silico primers obtained from http://www.staphylococcus.net/.

SNP calling and phylogenetic analysis
Paired end reads were mapped against the chromosome of *S. aureus* strain USA300-TCH1516 (NC_010079.1) (10) and N315 (NC_002745.2) using BWA v0.7.13 (11). Duplicate reads were identified using Picard Tools (https://broadinstitute.github.io/picard/) and ignored. SNP calling was performed using Pilon (https://www.broadinstitute.org/gaag/pilon) (12). Reads with a low quality score (<30), low coverage (<5 reads) or ambiguous SNPs from heterogeneous mappings were discarded. A maximum-likelihood tree was generated using RAxML v8.2.2 (13) assuming a General Time Reversible model under the gamma model of rate heterogeneity and with 1000 bootstrap replicates. A *S. aureus* strain from ST152 (NZ_LN854556.1) was utilized as a divergent outgroup to root the tree.

The Mesquite v3.2 algorithm (14) was used to estimate acquisition or loss of the SCC*mec* cassette by parsimony. To obtain accurate ancestry sorting, recombination blocks within each ST group were removed based on elevated SNP densities as per the gubbins algorithm (15). Whole genome maximum likelihood trees were built for each ST5 and ST8 using RAxML as described above.

Supplementary references

1. **Kim S**, **De Jonghe J**, **Kulesa AB**, **Feldman D**, **Vatanen T**, **Bhattacharyya RP**, **Berdy B**, **Gomez J**, **Nolan J**, **Epstein S**, **Blainey PC**. 2017. High-throughput automated microfluidic sample preparation for accurate microbial genomics. Nat Commun **8**:13919.

2. **Bankevich A**, **Nurk S**, **Antipov D**, **Gurevich AA**, **Dvorkin M**, **Kulikov AS**, **Lesin VM**, **Nikolenko SI**, **Pham S**, **Prjibelski AD**, **Pyshkin AV**, **Sirotkin AV**, **Vyahhi N**, **Tesler G**, **Alekseyev MA**, **Pevzner PA**. 2012. SPAdes: a new genome assembly algorithm and its applications to single-cell sequencing. J Comput Biol **19**:455–477.

3. **Jolley KA**, **Maiden MCJ**. 2010. BIGSdb: Scalable analysis of bacterial genome variation at the population level. BMC Bioinformatics **11**:595–595.

4. **Feil EJ**, **Li BC**, **Aanensen DM**, **Hanage WP**, **Spratt BG**. 2004. eBURST: inferring patterns of evolutionary descent among clusters of related bacterial genotypes from multilocus sequence typing data. Journal of Bacteriology **186**:1518–1530.

5. **Assefa S**, **Keane TM**, **Otto TD**, **Newbold C**, **Berriman M**. 2009. ABACAS: algorithm-based automatic contiguation of assembled sequences. Bioinformatics **25**:1968–1969.

6. **Seemann T**. 2014. Prokka: rapid prokaryotic genome annotation. Bioinformatics **30**:2068–2069.

7. **Darling AE**, **Mau B**, **Perna NT**. 2010. progressiveMauve: multiple genome alignment with gene gain, loss and rearrangement. PLoS ONE **5**:e11147.

8. **Altschul SF**, **Gish W**, **Miller W**, **Myers EW**, **Lipman DJ**. 1990. Basic local alignment search tool. J Mol Biol **215**:403–410.

9. **Carver TJ**, **Rutherford KM**, **Berriman M**, **Rajandream M-A**, **Barrell BG**, **Parkhill J**. 2005. ACT: the Artemis Comparison Tool. Bioinformatics **21**:3422–3423.

10. **Highlander SK**, **Hultén KG**, **Qin X**, **Jiang H**, **Yerrapragada S**, **Mason EO**, **Shang Y**, **Williams TM**, **Fortunov RM**, **Liu Y**, **Igboeli O**, **Petrosino J**, **Tirumalai M**, **Uzman A**, **Fox GE**, **Cardenas AM**, **Muzny DM**, **Hemphill L**, **Ding Y**, **Dugan S**, **Blyth PR**, **Buhay CJ**, **Dinh HH**, **Hawes AC**, **Holder M**, **Kovar CL**, **Lee SL**, **Liu W**, **Nazareth LV**, **Wang Q**, **Zhou J**, **Kaplan SL**, **Weinstock GM**. 2007. Subtle genetic changes enhance virulence of methicillin resistant and sensitive Staphylococcus aureus. BMC Microbiol **7**:99.

11. **Li H**, **Durbin R**. 2009. Fast and accurate short read alignment with Burrows-Wheeler transform. Bioinformatics **25**:1754–1760.

12. **Walker BJ**, **Abeel T**, **Shea T**, **Priest M**, **Abouelliel A**, **Sakthikumar S**, **Cuomo CA**, **Zeng Q**, **Wortman J**, **Young SK**, **Earl AM**. 2014. Pilon: an integrated tool for comprehensive microbial variant detection and genome assembly improvement. PLoS ONE **9**:e112963.

13. **Stamatakis A**. 2014. RAxML version 8: a tool for phylogenetic analysis and post-analysis of large phylogenies. Bioinformatics **30**:1312–1313.

14. **Maddison WP**, **Maddison DR**. Mesquite. mesquiteprojectorg, 2nd ed.

15. **Croucher NJ**, **Page AJ**, **Connor TR**, **Delaney AJ**, **Keane JA**, **Bentley SD**, **Parkhill J**, **Harris SR**. 2015. Rapid phylogenetic analysis of large samples of recombinant bacterial whole genome sequences using Gubbins. Nucleic Acids Research **43**:e15–e15.
